# miR-21-5p Alleviates Retinal Ischemia–Reperfusion Injury by Inhibiting M1 Polarization of Microglia via Suppression of STAT3 Signaling

**DOI:** 10.3390/biomedicines13102456

**Published:** 2025-10-09

**Authors:** Liangshi Qin, Junle Liao, Cheng Tan, Can Liu, Wenjia Shi, Dan Chen

**Affiliations:** Department of Human Anatomy and Neurobiology, School of Basic Medical Sciences, Central South University, Changsha 410013, China

**Keywords:** Retinal ischemia/reperfusion, oxygen–glucose deprivation, microglia, retinal ganglion cells, miR-21-5p, STAT3, retina, nerve injury, acute ocular hypertension, miRNA

## Abstract

**Background/Objectives**: Retinal ischemia–reperfusion (I/R) injury is a common mechanism in glaucoma, diabetic retinopathy, and retinal vein occlusion, leading to progressive loss of retinal ganglion cells (RGCs). This study investigates the regulatory role of miR-21-5p and its interaction with Signal Transducer and Activator of Transcription 3 (STAT3) in retinal I/R injury. **Methods**: An acute intraocular hypertension (AIH) rat model was used to induce retinal I/R. The interaction between miR-21-5p and STAT3 was examined by dual-luciferase reporter assays. miR-21-5p and STAT3 expression were quantified by qRT-PCR and Western blotting. Retinal morphology, microglial polarization, and RGC survival were assessed by H&E staining and immunofluorescence. In vitro, microglia and RGCs were subjected to oxygen–glucose deprivation/reperfusion (OGD/R), and microglial-conditioned media (MCM) were applied to RGCs. **Results**: (1) miR-21-5p ameliorated AIH-induced retinal damage in vivo. (2) Overexpression of miR-21-5p inhibits M1 polarization of RM cultured in vitro. (3) MCM from miR-21-5p-overexpressing microglia attenuated OGD/R-induced RGC death. (4) miR-21-5p downregulates STAT3 expression to inhibit RM M1 polarization. (5) miR-21-5p down-regulation of STAT3 levels inhibits M1 polarization and reduces apoptosis of RGCs in retinal microglia of AIH rats. **Conclusions**: miR-21-5p alleviates retinal I/R injury by restraining microglial M1 polarization through direct repression of STAT3, thereby promoting RGC survival. These findings identify the miR-21-5p/STAT3 axis as a potential therapeutic target for ischemic retinal diseases.

## 1. Introduction

Retinal ischemia–reperfusion (I/R) leads to loss of retinal ganglion cells (RGCs) and neuroinflammation, which is an important pathophysiological basis for various retinal diseases like glaucoma, diabetic retinopathy, and retinal vein occlusion, potentially leading to irreversible vision loss or blindness [1,2]. Neuroinflammation is a key driver of ischemic retinal pathology, wherein glial cells respond to injury by producing inflammatory factors that adversely affect RGC function and viability [3,4,5].

Microglia, the resident macrophages of the central nervous system (CNS), maintain homeostasis and, upon activation, release inflammatory mediators that drive optic nerve inflammation [6,7]. Microglia can polarize into either M1 or M2 phenotypes depending on external stimuli. M1 polarization, typically induced by lipopolysaccharide (LPS) or interferon-γ (IFN-γ), activates Toll-like receptor signaling and promotes the release of pro-inflammatory cytokines, nitric oxide (NO), and reactive oxygen species (ROS), ultimately leading to neuronal injury [8,9,10,11]. M2 polarization is typically induced by interleukin-4 (IL-4) or interleukin-13 (IL-13) and is characterized by the upregulation of anti-inflammatory mediators such as IL-10 and reparative factors including Arg-1, which contribute to extracellular matrix (ECM) remodeling, debris clearance, and tissue repair [12,13]. Previous studies have demonstrated that retinal I/R injury drives M1 polarization of retinal microglia, which in turn amplifies retinal inflammation and contributes to the loss of RGCs [14]. Therefore, elucidating the regulatory mechanisms of microglial polarization and developing strategies to suppress M1 polarization under I/R conditions are crucial for preventing retinal inflammation and treating ischemic retinal diseases.

MicroRNAs (miRNAs) are endogenous single-stranded non-coding RNAs that regulate gene expression by binding to the 3′ untranslated regions (3′-UTRs) of target mRNAs, thereby participating in diverse biological processes including apoptosis, inflammation, and cell polarization [15,16,17]. miR-21-5p, one of the earliest identified microRNAs, is markedly upregulated in models of neural injury and has been shown to play a pivotal regulatory role in the progression of neurological diseases. In traumatic nerve injury, neuronal extracellular vesicles are enriched with miR-21-5p, which promotes neuroprotection by inhibiting autophagy, enhancing neuronal survival, supporting axonal growth, and facilitating regeneration [18,19,20,21,22]. In retinal I/R injury and glaucoma models, miR-21-5p has been reported to facilitate aqueous humor outflow by activating the eNOS/MMP9 signaling pathway in trabecular meshwork (TM) and Schlemm’s canal (SC) cells, thereby lowering intraocular pressure [23]. Furthermore, miR-21-5p has been shown to attenuate I/R-induced pyroptosis of RGCs by inhibiting activation of the NLRP3/Caspase-1 pathway, thereby mitigating inflammation and preserving visual function [4,24]. Moreover, evidence indicates that miR-21-5p facilitates M2 polarization of macrophages by targeting PDCD4 and modulating the SPRY2/ERK signaling pathway [25,26]. Although miR-21-5p has been implicated in alleviating retinal I/R injury [27], the underlying mechanisms remain insufficiently defined. In particular, whether it exerts protective effects by modulating retinal microglial polarization and thereby regulating neuroinflammation has not been systematically examined.

This study established rat models of acute intraocular hypertension (AIH) and cellular oxygen–glucose deprivation/reperfusion (OGD/R) to investigate miR-21-5p expression in retina and rat microglia (RM). TargetScan prediction and dual-luciferase reporter assays identified *Signal Transducer and Activator of Transcription 3 (STAT3)* as a direct target of miR-21-5p. STAT3 is a transcription factor that regulates proliferation, survival, differentiation, and metabolism [28]. Upon activation, STAT3 induces the expression of cell cycle- and survival-related genes, including *Cyclin D1*, *Survivin*, and *c-Myc*, while driving the transcription of downstream inflammation-related genes such as *IL-6*, *IL-1β*, *TNF-α*, and *COX-2*, thereby amplifying inflammatory cascades during I/R injury [28,29,30,31]. STAT3 activation is regulated by phosphorylation, which is markedly elevated under I/R conditions; phosphorylated STAT3 (p-STAT3) translocates into the nucleus and promotes the transcription of downstream target genes, thereby amplifying inflammatory responses and aggravating tissue injury [32,33,34]. Inhibition of STAT3 signaling has been demonstrated to attenuate neuronal injury and restrain macrophage activation [35], thereby identifying STAT3 as a promising therapeutic target in I/R.

To test causality, RM were transfected with an miR-21-5p mimic or treated with the STAT3 inhibitor S3I-201, and, in vivo, rats received intravitreal delivery of an miR-21-5p agomir or S3I-201 before AIH-induced retinal I/R. These complementary gain- and loss-of-function manipulations demonstrate that miR-21-5p alleviates retinal I/R injury by restraining STAT3-dependent pro-inflammatory signaling, nominating the miR-21-5p/STAT3 axis as a potential therapeutic target.

## 2. Materials and Methods

### 2.1. Rat Model of AIH

Male Sprague Dawley rats (6–8 weeks; 200–250 g) obtained from the Experimental Animal Centre of Central South University were used. Animals were housed in a temperature- and humidity-controlled room on a 12 h light/dark cycle with ad libitum access to food and water.

Rats were randomly divided into two groups: the normal control group (without I/R surgery) and the AIH group. The acute intraocular hypertension (AIH) model was established as described previously [36]. Rats were anesthetized via intraperitoneal injection of 2% pentobarbital sodium (0.2 mL/100 g). The periocular skin was cleaned with povidone–iodine, followed by topical anesthesia with proparacaine ophthalmic solution and pharmacologic mydriasis with tropicamide/phenylephrine. Intraocular pressure was raised to 120 mmHg for 1 h via anterior chamber cannulation with a 30-gauge saline-connected needle under general anesthesia, followed by reperfusion; rats were euthanized 3 days post-reperfusion.

### 2.2. Intravitreal Injection

A total of 40 male SD rats (6–8 weeks old, 200–250 g) were randomly assigned to five groups (n = 8 per group): normal control (CON), AIH, miR-21-5p agomir+AIH, miR-21-5p agomir negative control (NC)+AIH, and S3I-201+AIH.

miR-21-5p agomir (chemically modified miR-21-5p mimic, 2 µL at 50 µM per eye) and its negative control (scrambled sequence) were synthesized by Sangon Biotech (Shanghai, China). S3I-201(0.2 mg/kg) was purchased from Selleck Chemicals (Houston, TX, USA). Intravitreal injections were performed 30 min before AIH induction. Injections were made 1 mm posterior to the limbus between two vortex veins using a 30-gauge needle, as described previously [37].

### 2.3. Tissue Preparation

Following euthanasia, both globes were enucleated and fixed in 4% paraformaldehyde (PFA) at 4 °C for 24 h, followed by graded ethanol dehydration (70%, 80%, 90%, 95%, and 100%; 1 h each), clearing in ethanol–xylene and xylene, and paraffin infiltration according to standard histological protocols [38]. Paraffin blocks were sectioned at 5 µm, mounted on glass slides, labeled, and stored at room temperature (RT). Retinal sections adjacent to the optic nerve head were used for subsequent histological analyses.

In a separate cohort, retinas were rapidly dissected after euthanasia, placed into pre-chilled microcentrifuge tubes on ice, and stored at −80 °C until use.

### 2.4. Cell Culture and Transfection

Rat microglia (RM; BNCC360237) were obtained from BeNa Culture Collection (BeNa Culture Collection, Beijing, China). Rat retinal precursor cells (R28) were obtained from the Affiliated Eye Hospital of Nanchang University (Nanchang, China). RM were maintained in high-glucose DMEM (Gibco) supplemented with 10% fetal bovine serum (FBS, Gibco). R28 cells were cultured in low-glucose DMEM (Gibco) containing 10% FBS. All cells were incubated at 37 °C in a humidified atmosphere of 5% CO_2_.

RM at 70–80% confluence were transfected using Advanced Series High-Efficiency Transfection Reagent with miR-21-5p mimic (50 nM) or mimic NC (50 nM); for gain-of-function, a STAT3 overexpression plasmid (Shanghai Bioengineering Co., Ltd., Shanghai, China) was used. S3I-201 (30 µM) was applied for pharmacologic inhibition. Vendors for the miRNA reagents and S3I-201 are as described above. Cells were harvested 24 h after treatment.

### 2.5. Oxygen–Glucose Deprivation/Reperfusion (OGD/R) and Conditioned Medium Transfer

RM and R28 cells were switched to glucose-free DMEM (Gibco) and placed in a hypoxia chamber (1% O_2_, 5% CO_2_, 94% N_2_) at 37 °C for 4 h (RM) or 3 h (R28). After OGD, cultures were returned to normoxia (humidified 5% CO_2_ at 37 °C) for reperfusion. Microglia-conditioned media (MCM; CON, OGD/R, miR-21-5p mimic NC+OGD/R, miR-21-5p mimic+OGD/R) were collected from RM after the indicated treatments and immediately applied to R28 at the end of OGD; R28 were then incubated with the indicated MCM for 2 h under standard culture conditions.

### 2.6. Real-Time Quantitative PCR (qRT-PCR)

Total RNA was isolated using TRIzol (TSP401; DynaTech Biotechnology, Shanghai, China). For miRNA quantification, miR-21-5p was reverse-transcribed with a stem-loop RT primer; for mRNA analysis, STAT3 and β-actin (Actb) were reverse-transcribed using the RevertAid First Strand cDNA Synthesis Kit (K1622; Thermo Fisher Scientific, Waltham, MA, USA). Primers (miR-21-5p stem-loop/forward/reverse; U6; Actb; STAT3) were synthesized by DynaProbiotics (Shanghai, China). U6 served as the endogenous control for miRNA assays, and β-actin served as the endogenous control for mRNA assays. Relative expression was calculated by the 2^^−ΔΔCt^ method. Primer sequences (5′→3′): miR-21-5p, stem-loop RT: GTCGTATCCAGTG-CAGGGTCCGAGGTATTCGCACTGGATAC-GACTCAACA, forward: GCGCGTAGCTTATCAGACTGA, reverse: AGTGCAGGGTCCGAGGTATT; U6, forward: CTGTGGAGAAGGGAGGGTGAGAG, reverse: AGGTGAGAAGGAGGTGCAGACTG; β-actin (Actb), forward: TACTGCCCTGGCTCCTAGCA, reverse: TGGACAGTGAGGCCAGGATAG; STAT3, forward: AGGGCTTCTCGTTCTGGGTCTG, reverse: CTCCCGCTCCTTGCTGATGAAAC; IL-1β, forward: GACTTCACCATGGAACCCGT, reverse: GGAGACTGCCCATTCTCGAC; TNF-α, forward: CATCCGTTCTCTACCCAGCC, reverse: AATTCTGAGCCCGGAGTTGG; IL-6, forward: GCCCACCAGGAACGAAAGTC, reverse: ACTGGCTGGAAGTCTCTTGCG.

### 2.7. Hematoxylin and Eosin (H&E) Staining

Paraffin-embedded retinal sections (5 µm) were deparaffinized in xylene (2 × 10 min), rehydrated through graded ethanol (100%, 95%, 70%, 5 min each) to water, stained with hematoxylin (5 min) and eosin (2 min), then dehydrated through 95% and 100% ethanol (5 min each), cleared in xylene (2 × 5 min) and coverslipped [39]. Images were acquired under a light microscope (Nikon, Tokyo, Japan), and retinal morphology was evaluated in a blinded manner.

### 2.8. Immunofluorescence

#### 2.8.1. Immunofluorescence of Retinal Paraffin Sections

Deparaffinization and rehydration were performed as described in Section 2.7 (H&E), followed by transfer to distilled water. Sections were then subjected to heat-induced epitope retrieval in sodium citrate buffer (pH 6.0) and blocked with 5% goat serum for 1 h at RT [40]. Sections were incubated overnight at 4 °C with primary antibodies: Iba1 (mouse, 1:1000, Huabio, Hangzhou, China, Cat# RT1316, RRID: AB_3698022), CD206 (rabbit, 1:500, Proteintech, Wuhan, China, Cat# 18704-1-AP, RRID: AB_10597232), CD86 (rabbit, 1:500, Proteintech, Cat# 83523-4-RR, RRID: AB_3671150), and RBPMS (rabbit, 1:500, Abcam, Cambridge, UK, Cat# ab152101, RRID: AB_2923082). After PBS washes (3 × 5 min), sections were incubated 1 h at RT with FITC-conjugated goat anti-mouse IgG (Servicebio, Wuhan, China, Cat# GB21303) and Cy3-conjugated goat anti-rabbit IgG (Servicebio, Cat# GB22301). Nuclei were counterstained with DAPI (10 µL, 5 min, RT), and sections were mounted with antifade mounting medium (Beyotime, Shanghai, China, Cat# P0126). Images were acquired on a Nikon fluorescence microscope. Microglial polarization was quantified as the percentage of Iba1^+^ cells co-expressing CD86 (M1) or CD206 (M2). Fluorescence intensity and cell counts were measured with ImageJ (NIH, Bethesda, MD, USA). At least three fields per coverslip and three independent experiments were analyzed.

#### 2.8.2. Cellular Immunofluorescence

Cells were washed with PBS, fixed in 4% PFA for 20 min, washed again with PBS, permeabilized with 0.5% Triton X-100 for 20 min, and blocked with 5% goat serum for 1 h at RT [41]. Cells were incubated overnight at 4 °C with CD206 and CD86 (same primary antibodies and dilutions as in Section 2.8.1), followed by the same secondary antibodies, DAPI counterstain, and mounting procedures as described above.

### 2.9. TUNEL Staining

Apoptosis in retinal sections and R28 cells was assessed using a TUNEL Apoptosis Detection Kit (Beyotime, Shanghai, China, Cat# C1090) according to the manufacturer’s instructions. Nuclei were counterstained with DAPI. Images were acquired on a Nikon fluorescence microscope. TUNEL-positive cells and total nuclei (DAPI) were quantified per field by blinded observers using ImageJ (NIH, Bethesda, MD, USA), and the percentage of apoptotic cells was calculated as TUNEL^+^/DAPI × 100%.

### 2.10. PI Staining

Cells were fixed in 4% PFA for 15 min, washed with PBS, and incubated with propidium iodide (PI; Sigma-Aldrich, Darmstadt, Germany, Cat# P4170) working solution for 15 min protected from light. After PBS washes, cells were counterstained with DAPI (5 min, RT), mounted with antifade medium, and imaged using a Nikon fluorescence microscope [42]. PI-positive cells and total nuclei were quantified with ImageJ by blinded observers.

### 2.11. Western Blotting (WB)

Retinal tissues were homogenized on ice in RIPA buffer (Beyotime, Shanghai, China, P0013B) supplemented with protease and phosphatase inhibitors (Cwbio, Beijing, China, CW2200S; CW2383S), centrifuged (12,000× *g*, 20 min, 4 °C), and the supernatants were collected for protein quantification by BCA (Beyotime, P0010S). Cultured cells were washed with ice-cold PBS, lysed on ice in the same RIPA buffer with inhibitors, centrifuged under identical conditions, and supernatants were quantified by BCA. Equal amounts of protein (20 µg per lane) were resolved on 10% SDS–PAGE and transferred to PVDF membranes (Thermo Fisher Scientific, Waltham, MA, USA). Membranes were blocked with 5% nonfat milk for 1 h at room temperature (RT), incubated with primary antibodies overnight at 4 °C, washed with TBST (3 × 10 min), and then incubated with HRP-conjugated goat anti-rabbit or goat anti-mouse secondary antibodies (1:4000; Jackson ImmunoResearch, West Grove, PA, USA, 315-005-003 and 211-009-109) for 2 h at RT. Signals were developed by chemiluminescence and quantified with ImageJ; target proteins were normalized to actin and expressed relative to control.

Primary antibodies: CD206 (Rabbit, 1:1000, Proteintech, Wuhan, China, Cat# 18704-1-AP, RRID: AB_10597232), CD86 (Rabbit, 1:2000, Huabio, Hangzhou, China Cat# ET1606-50, RRID: AB_3069745), Arg-1 (Rabbit, 1:5000, Proteintech, Wuhan, China, Cat# 16001-1-AP, RRID: AB_2289842), IL-1β (Rabbit, 1:2000, G-Biosciences, St. Louis, MO, USA, Cat# ITT5201, RRID: AB_3662796), Bcl-2 (Rabbit, 1:500, WanLeiBio, Shenyang, China, Cat# WL01556, RRID: AB_2904235), Bax (Rabbit, 1:500, WanLeiBio Cat# WL01637, RRID: AB_2904236), STAT3 (Rabbit, 1:1000, WanLeiBio Cat# WL01836, RRID: AB_3665484), Phospho-Stat3 (Rabbit, 1:2000, Cell Signaling Technology, Danvers, MA, USA, Cat# 73533, RRID: AB_3675996), actin (Mouse, 1:10000, Cohesion Biosciences, London, UK, Cat# CPA9100, RRID: AB_3697272), TNF-α (Rabbit, 1:2000, Proteintech Cat# 80258-6-RR, RRID: AB_3670472), IL-6 (Rabbit, 1:2000, Proteintech Cat# 21865-1-AP, RRID: AB_11142677).

### 2.12. Dual-Luciferase Reporter Assay

HEK293T cells were co-transfected with STAT3 3′-UTR reporter plasmids—wild type (WT) or mutant (MUT)—together with miR-21-5p mimic or mimic negative control (NC). After 24 h, luciferase activity was measured using the Dual-Luciferase Reporter Assay System (Promega, Madison, WI, USA). Firefly luciferase signals were normalized to Renilla luciferase to control for transfection efficiency. Data are presented as relative luciferase activity (Firefly/Renilla). Each condition was tested in technical triplicates, and the experiment was repeated in at least three independent biological replicates.

### 2.13. Statistical Analysis

Data are expressed as mean ± SD. For comparisons between two groups, unpaired two-tailed *t*-tests were used. For ≥3 groups, one-way ANOVA followed by the Bonferroni post hoc test was performed. n denotes independent biological replicates (typically *n* = 3 per group; technical triplicates where indicated). Analyses were conducted in GraphPad Prism 9.0 (GraphPad Software, San Diego, CA, USA). Statistical significance was set at *p* < 0.05.

## 3. Results

### 3.1. miR-21-5p Ameliorated AIH-Induced Retinal Damage In Vivo

**Figure 1:**
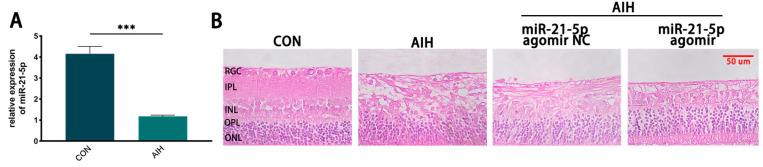
miR-21-5p alleviates AIH-induced retinal damage in rats. (**A**) qRT–PCR analysis of retinal miR-21-5p under AIH; expression was significantly reduced versus CON (*** *p* < 0.001; unpaired two-tailed *t*-test; n = 3; data = mean ± SD). (**B**) H&E staining of rat retina. Scale bar = 50 μm. AIH and miR-21-5p agomir NC + AIH showed marked structural disruption relative to CON, whereas miR-21-5p agomir + AIH exhibited attenuated histopathological damage.

Using an AIH model to induce retinal I/R injury, intraocular pressure was elevated to 120 mmHg for 1 h. Rats were euthanized 3 days after reperfusion, and retinal miR-21-5p levels were quantified by qRT–PCR. Compared with the CON group, miR-21-5p expression was significantly decreased in AIH retinas (*p* < 0.001; Figure 1A). H&E staining showed marked retinal structural damage in the AIH group, which was mitigated by pretreatment with miR-21-5p agomir (miR-21-5p agomir + AIH; Figure 1B). Collectively, these data indicate that miR-21-5p exerts a protective effect in AIH-induced retinal I/R injury.

### 3.2. Overexpression of miR-21-5p Inhibits M1 Polarization of RM Cultured In Vitro

**Figure 2:**
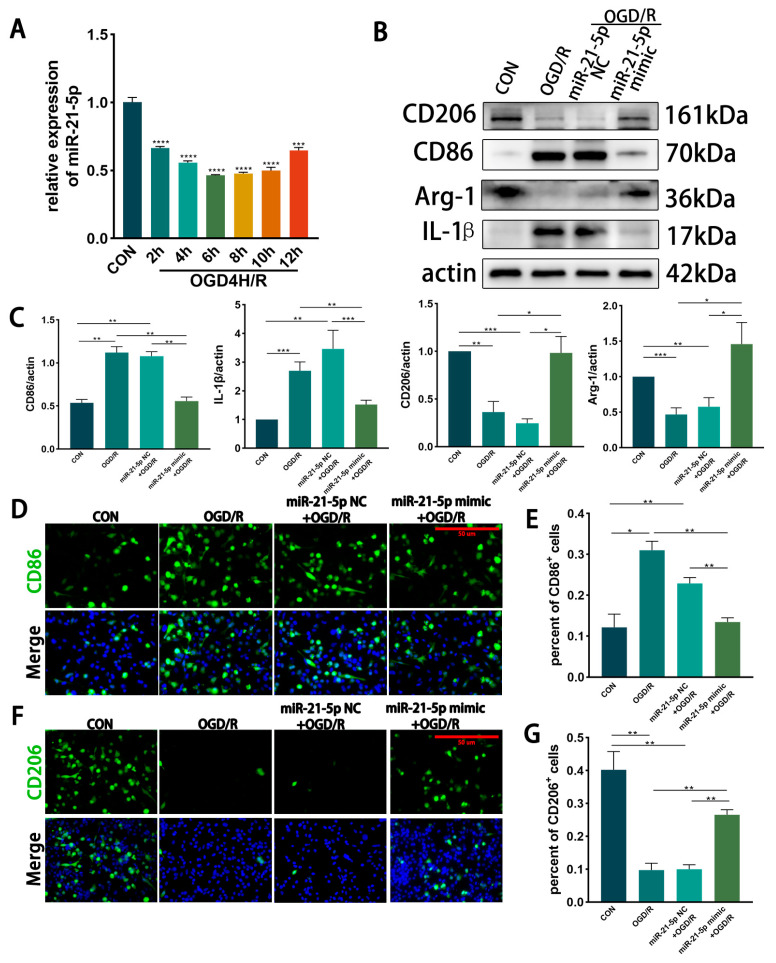
Overexpression of miR-21-5p inhibits M1 polarization of RM under OGD/R conditions. (**A**) qRT-PCR analysis of miR-21-5p expression in RM at different recovery time points after 4 h OGD. Expression decreased progressively, reaching the lowest levels at 6 h and 8 h, and partially rebounded at 12 h but remained below CON (*** *p* < 0.001, **** *p* < 0.0001; one-way ANOVA; *n* = 3). (**B**,**C**) WB detection and densitometric quantification of polarization-associated proteins. Compared with CON, the OGD/R and miR-21-5p NC + OGD/R groups showed elevated CD86 and IL-1β and reduced CD206 and Arg-1. In contrast, the miR-21-5p mimic + OGD/R group exhibited reduced CD86 and IL-1β and increased CD206 and Arg-1 (* *p* < 0.05, ** *p* < 0.01, *** *p* < 0.001; one-way ANOVA; *n* = 3). (**D**–**G**) Immunofluorescence analysis of RM polarization. (**D**,**E**) CD86^+^ RM (M1-like, green) and nuclei (DAPI, blue). (**F**,**G**) CD206^+^ RM (M2-like, green) and nuclei (DAPI, blue). Scale bar = 50 μm. Quantification shows a higher proportion of CD86^+^ and a lower proportion of CD206^+^ RM in the OGD/R and miR-21-5p NC + OGD/R groups compared with CON. The miR-21-5p mimic + OGD/R group displayed a lower percentage of CD86^+^ RM and a higher percentage of CD206^+^ RM relative to both OGD/R controls (* *p* < 0.05, ** *p* < 0.01; one-way ANOVA; *n* = 3).

In vitro time course of miR-21-5p under OGD/R. RM were subjected to OGD for 4 h followed by reoxygenation, and miR-21-5p expression was measured at different recovery time points by qRT-PCR. Expression levels progressively decreased during reoxygenation, reaching a nadir at 6 h and 8 h (*p* < 0.001 vs. CON). By 12 h, expression partially rebounded but remained significantly lower than control levels (Figure 2A). These in vitro kinetics were consistent with the in vivo findings. Based on this profile, we selected OGD4 h/R6 h for subsequent experiments, as it represented the time point with the most pronounced reduction.

Effect of miR-21-5p overexpression on RM polarization under OGD/R. RM cells were transfected with miR-21-5p mimics, with scramble mimics serving as negative controls. After OGD4 h/R6 h, WB and immunofluorescence analyses were performed. Compared with CON, both the OGD/R and miR-21-5p NC + OGD/R groups showed markedly increased expression of the M1 marker CD86 and the pro-inflammatory cytokine IL-1β, along with reduced expression of the M2 marker CD206 and the anti-inflammatory factor Arg-1 (Figure 2B,C). In contrast, the miR-21-5p mimic + OGD/R group exhibited significantly lower protein levels of CD86 and IL-1β while CD206 and Arg-1 expression were upregulated (Figure 2B,C).

Immunofluorescence results further confirmed that the proportion of CD86^+^ (M1-like) RM was higher and the proportion of CD206^+^ (M2-like) RM was lower in the OGD/R and miR-21-5p NC + OGD/R groups compared with CON, whereas the miR-21-5p mimic + OGD/R group displayed a lower percentage of CD86^+^ RM and a higher percentage of CD206^+^ RM relative to both OGD/R controls (Figure 2D–G). Together, these results demonstrate that miR-21-5p overexpression inhibits M1 polarization while promoting M2 polarization of RM under OGD/R conditions, thereby downregulating pro-inflammatory cytokines and upregulating anti-inflammatory factors.

### 3.3. MCM from miR-21-5p-Overexpressing Microglia Attenuated OGD/R-Induced R28 Death

Building on the observation that miR-21-5p suppresses M1 polarization of RM under OGD/R, we next examined its impact on neuronal survival. R28 cells were subjected to OGD for 3 h [43], after which the culture medium was replaced with MCM collected from the groups described in Result 2 (CON, OGD/R, miR-21-5p NC + OGD/R, and miR-21-5p mimic + OGD/R), followed by 2 h of reoxygenation.

**Figure 3:**
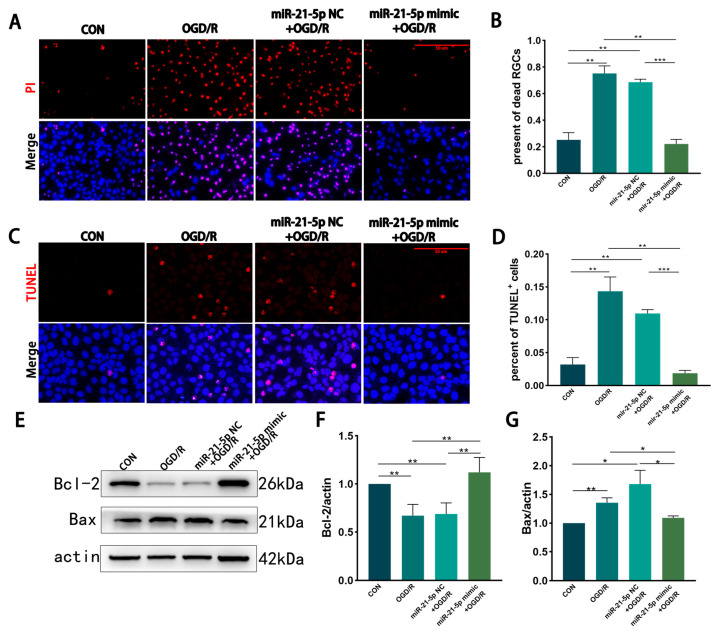
RM-conditioned medium with miR-21-5p overexpression under OGD/R conditions promotes R28 cells survival in vitro. (**A**,**B**) PI staining showing necrosis of R28 cells after OGD (3 h) and reoxygenation (2 h) in different RM-conditioned media. PI (red), nuclei (DAPI, blue). Scale bar = 50 μm. Quantification indicates increased PI^+^ cells in the OGD/R and miR-21-5p NC + OGD/R groups compared with CON, whereas the miR-21-5p mimic + OGD/R group showed fewer PI^+^ cells (** *p* < 0.01, *** *p* < 0.001; one-way ANOVA; *n* = 3). (**C**,**D**) TUNEL staining of apoptotic R28 cells. TUNEL (red), nuclei (DAPI, blue). Scale bar = 50 μm. Quantification shows elevated TUNEL^+^ cells in the OGD/R and miR-21-5p NC + OGD/R groups versus CON, while the miR-21-5p mimic + OGD/R group exhibited a lower proportion (** *p* < 0.01, *** *p* < 0.001; one-way ANOVA; *n* = 3). (**E**–**G**) WB analysis of apoptosis-related proteins. Representative blots and densitometry of Bcl-2 and Bax. Compared with CON, the OGD/R and miR-21-5p NC + OGD/R groups showed reduced Bcl-2 and increased Bax, whereas the miR-21-5p mimic + OGD/R group displayed higher Bcl-2 and lower Bax (* *p* < 0.05, ** *p* < 0.01; one-way ANOVA; *n* = 3).

PI and TUNEL staining revealed that, compared with MCM from CON RM, MCM from the OGD/R and miR-21-5p NC + OGD/R groups significantly increased necrosis and apoptosis of R28 cells (Figure 3A–D). In contrast, MCM from the miR-21-5p mimic + OGD/R group markedly reduced necrotic and apoptotic cell percentages compared with both OGD/R control groups (Figure 3A–D). WB analysis further showed that R28 cells exposed to MCM from the miR-21-5p mimic + OGD/R group displayed higher expression of the anti-apoptotic protein Bcl-2 and lower expression of the pro-apoptotic protein Bax than those treated with MCM from the OGD/R or miR-21-5p NC + OGD/R groups (Figure 3E–G). Collectively, these results indicate that RM overexpressing miR-21-5p alleviate OGD/R-induced necrosis and apoptosis in R28 cells, thereby exerting a neuroprotective effect.

### 3.4. miR-21-5p Downregulates STAT3 Expression to Inhibit RM M1 Polarization

In summary, overexpression of miR-21-5p under OGD/R conditions suppressed M1 polarization while promoting M2 polarization of RM, thereby reducing RGC necrosis and apoptosis. To clarify the mechanism, we next examined whether this effect involves STAT3 signaling.

#### 3.4.1. STAT3 Is the Direct Target Gene of miR-21-5p

**Figure 4:**
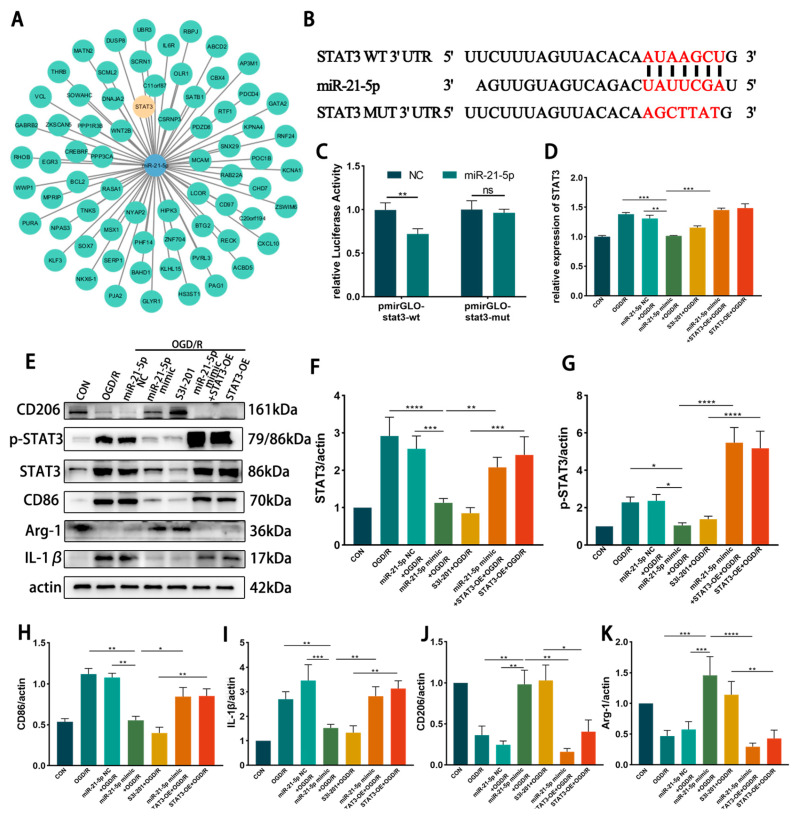

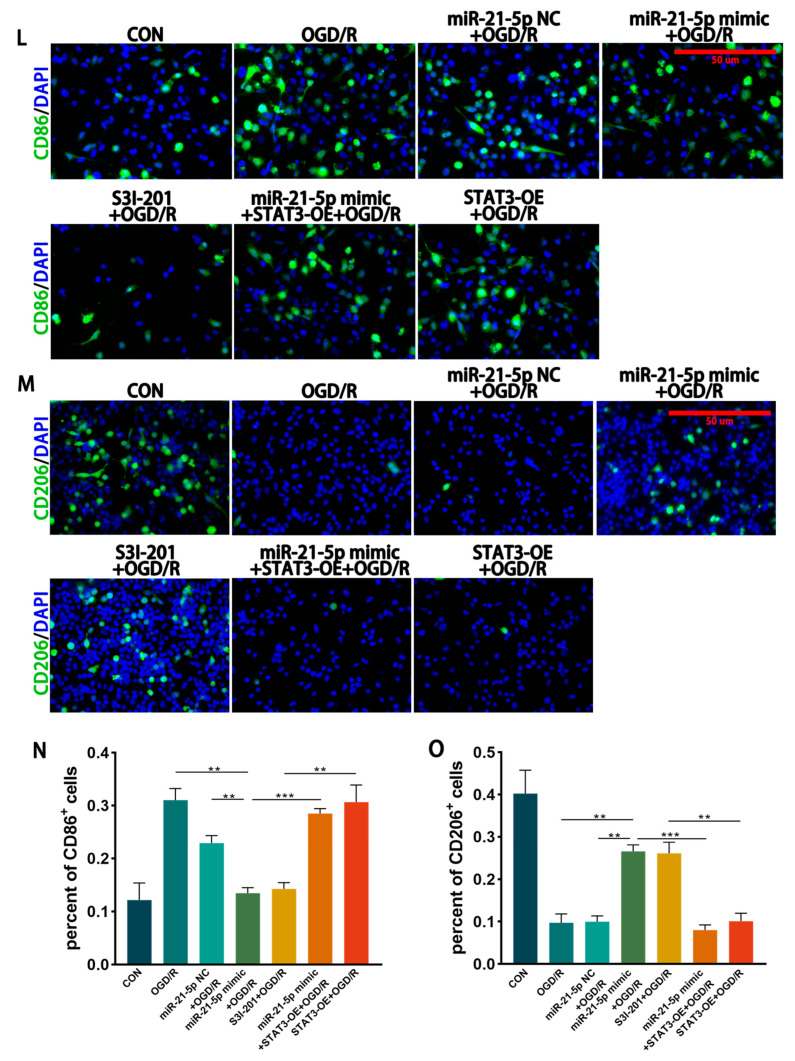
miR-21-5p directly targets STAT3 and regulates RM polarization under OGD/R conditions. (**A**) TargetScan prediction of miR-21-5p target genes. (**B**) Binding site between miR-21-5p and the STAT3 3′-UTR. (**C**) Dual-luciferase reporter assay confirming direct binding between miR-21-5p and STAT3 (** *p* < 0.01). (**D**) qRT-PCR analysis of STAT3 expression in RM (** *p* < 0.01, *** *p* < 0.001; one-way ANOVA, *n* = 3). STAT3 mRNA was reduced in the miR-21-5p mimic + OGD/R group compared with the OGD/R and NC groups, but increased with STAT3-OE co-transfection. (**E**–**K**) WB analysis and quantification of STAT3, p-STAT3, and polarization-related proteins (* *p* < 0.05, ** *p* < 0.01, *** *p* < 0.001, **** *p* < 0.0001; one-way ANOVA, *n* = 3). p-STAT3, STAT3, CD86, and IL-1β were decreased, whereas CD206 and Arg-1 were increased, in the miR-21-5p mimic + OGD/R and S3I-201 + OGD/R groups compared with OGD/R, NC, and STAT3-OE groups. (**L**–**M**) Immunofluorescence staining of RM polarization. (**L**) CD86 (green), DAPI (blue); (**M**) CD206 (green), DAPI (blue). Quantification (**N**,**O**) showed fewer CD86^+^ RM and more CD206^+^ RM in the miR-21-5p mimic + OGD/R and S3I-201 + OGD/R groups compared with OGD/R, NC, and STAT3-OE groups (** *p* < 0.01, *** *p* < 0.001; one-way ANOVA, *n* = 3).

**Figure A1:**
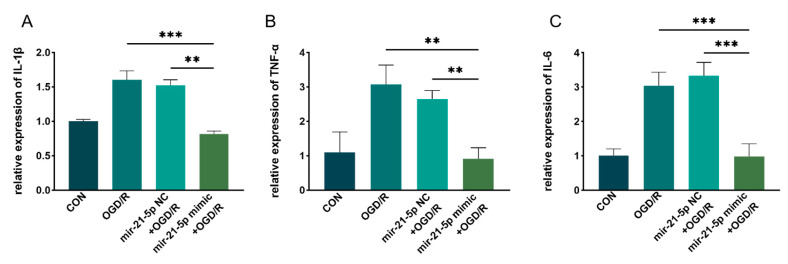
qRT–PCR analysis of STAT3 downstream inflammatory mediators in rat microglia (RM) under OGD/R with the indicated treatments. Relative mRNA levels of (**A**) IL-1β, (**B**) TNF-α, and (**C**) IL-6 were quantified across CON, OGD/R, miR-21-5p mimic NC + OGD/R, and miR-21-5p mimic + OGD/R groups. Values were normalized to β-actin and calculated by the 2^^−ΔΔCt^ method. Data are shown as mean ± SD (n = 3 independent experiments). Statistical comparisons were performed by one-way ANOVA followed by Bonferroni post hoc test; ** *p* < 0.01, *** *p* < 0.001 vs. indicated controls. These results show that miR-21-5p overexpression significantly reduces STAT3-responsive pro-inflammatory transcripts following OGD/R.

To explore potential downstream targets of miR-21-5p in RM, TargetScan analysis was conducted and predicted STAT3, a transcription factor linked to inflammation, as a candidate (Figure 4A). A dual-luciferase reporter assay confirmed direct binding between miR-21-5p and the 3′-UTR of STAT3 (*p* < 0.001; Figure 4B,C). qRT-PCR revealed that STAT3 mRNA levels were significantly decreased in the miR-21-5p mimic + OGD/R group compared with the OGD/R group and the NC + OGD/R group (Figure 4D). Consistently, WB analysis demonstrated that total STAT3, phosphorylated STAT3 (p-STAT3), and the downstream inflammatory protein IL-1β were markedly reduced in the miR-21-5p mimic + OGD/R group relative to both control groups (Figure 4E–G,I). Moreover, qRT-PCR showed decreased mRNA levels of STAT3 downstream inflammatory mediators, including IL-1β, TNF-α, and IL-6 (Figure A1). Collectively, these findings identify STAT3 as a direct functional target of miR-21-5p and demonstrate that miR-21-5p suppresses its expression and downstream pro-inflammatory signaling.

#### 3.4.2. Inhibition of STAT3 Reduces M1 Polarization of RM Cultured In Vitro

To further investigate the regulatory role of STAT3 in RM polarization in vitro, RM were transfected with either a STAT3 overexpression plasmid (STAT3-OE) or treated with the STAT3 inhibitor S3I-201, followed by induction of the OGD 4 h/R 6 h model. WB analysis showed that STAT3 and p-STAT3 protein levels were markedly reduced in the S3I-201 + OGD/R group compared with the OGD/R group, whereas STAT3 protein expression was not significantly altered in the STAT3-OE + OGD/R group (Figure 4E–G). WB results further demonstrated that, relative to the OGD/R and STAT3-OE + OGD/R groups, the S3I-201 + OGD/R group exhibited lower expression of CD86 and IL-1β, while CD206 and Arg-1 levels were increased (Figure 4E–K). Immunofluorescence confirmed these findings, showing decreased percentages of CD86^+^ RM and increased percentages of CD206^+^ RM in the S3I-201 + OGD/R group compared with controls (Figure 4L–O). Together, these results indicate that inhibition of STAT3 activity suppresses M1 polarization of RM and attenuates pro-inflammatory responses in vitro.

#### 3.4.3. miR-21-5p Downregulates STAT3 Levels to Inhibit M1 Polarization of RM

To determine whether miR-21-5p inhibits M1 polarization of RM by downregulating STAT3, RM were co-transfected with an miR-21-5p mimic and a STAT3 overexpression plasmid (STAT3-OE), followed by induction of the OGD 4 h/R 6 h model. qRT-PCR confirmed that STAT3 mRNA levels were significantly higher in the miR-21-5p mimic + STAT3-OE + OGD/R group than in the miR-21-5p mimic + OGD/R group (Figure 4D). WB analysis further showed that STAT3 overexpression reversed the effects of miR-21-5p, with increased CD86 and IL-1β expression and decreased CD206 and Arg-1 expression (Figure 4E–K). Immunofluorescence results were consistent, showing a higher percentage of CD86^+^ RM and a lower percentage of CD206^+^ RM in the co-transfection group compared with the miR-21-5p mimic group (Figure 4L–O). Together, these results demonstrate that miR-21-5p inhibits M1 polarization of RM and alleviates inflammatory responses by negatively regulating STAT3 expression.

### 3.5. miR-21-5p Down-Regulation of STAT3 Levels Inhibits M1 Polarization and Reduces Apoptosis of RGCs in Retinal Microglia of AIH Rats

**Figure 5:**
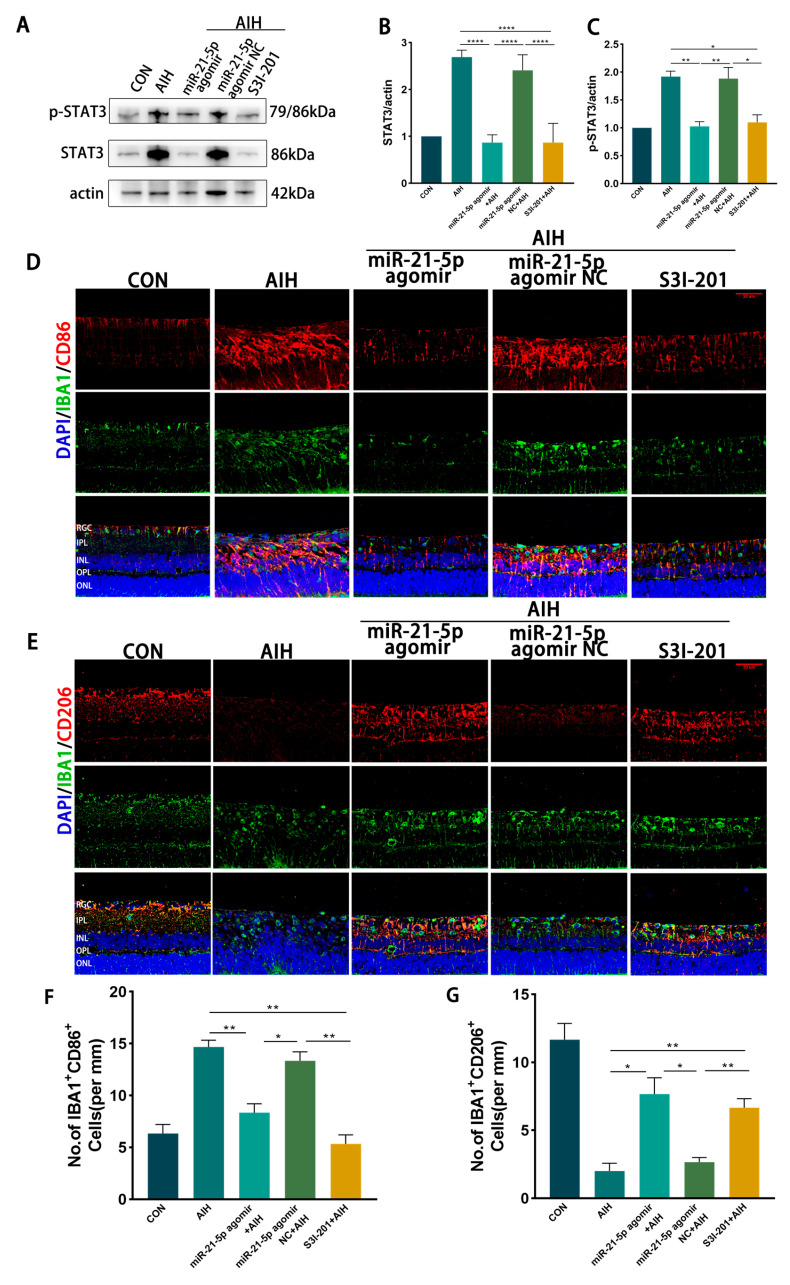

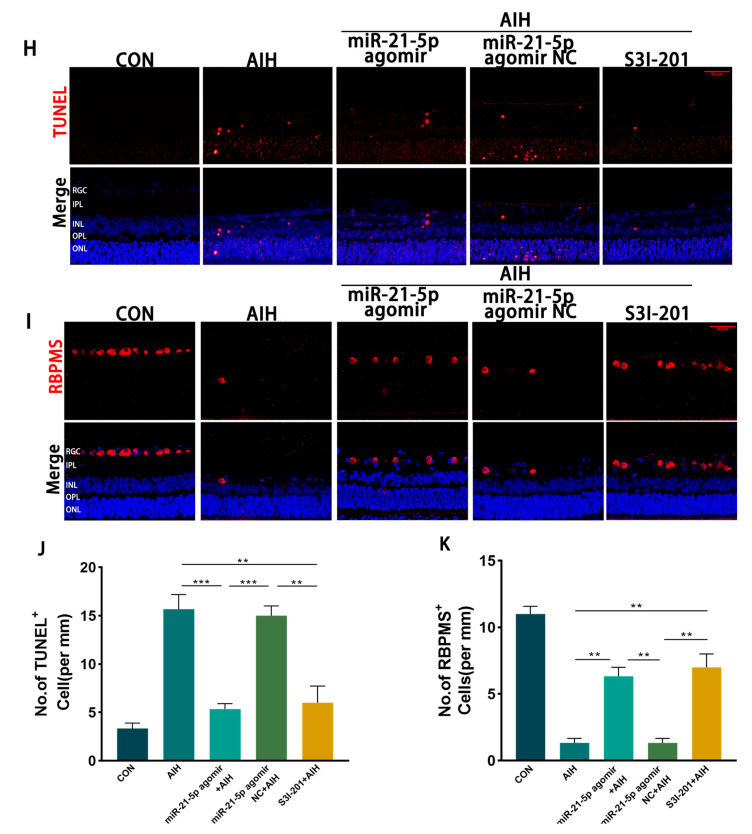
miR-21-5p downregulates STAT3 expression in vivo to inhibit M1 polarization of retinal microglia and reduce RGCs apoptosis. (**A**–**C**) WB analysis of STAT3 and p-STAT3 expression in rat retinas, with quantification (* *p* < 0.05, ** *p* < 0.01, **** *p* < 0.0001; one-way ANOVA, *n* = 3). STAT3 and p-STAT3 levels were elevated in the AIH and miR-21-5p agomir NC + AIH groups compared with CON, whereas both were reduced in the miR-21-5p agomir + AIH and S3I-201 + AIH groups. (**D**–**G**) Immunofluorescence staining of retinal microglial polarization. (**D**) IBA1 (green), CD86 (red), DAPI (blue); (**E**) IBA1 (green), CD206 (red), DAPI (blue). Quantification showed that IBA1^+^CD86^+^ (M1-like) microglia were increased and IBA1^+^CD206^+^ (M2-like) microglia were decreased in the AIH and NC groups compared with CON. In contrast, the miR-21-5p agomir + AIH and S3I-201 + AIH groups exhibited reduced numbers of IBA1^+^CD86^+^ and increased numbers of IBA1^+^CD206^+^ cells (* *p* < 0.05, ** *p* < 0.01; one-way ANOVA, *n* = 3). (**H**) TUNEL staining of RGC apoptosis (red), with DAPI (blue). The number of TUNEL^+^ RGCs was higher in the AIH and NC groups but reduced in the miR-21-5p agomir + AIH and S3I-201 + AIH groups. (**I**) RBPMS staining of RGC survival (red), with DAPI (blue). Compared with AIH and NC groups, RBPMS^+^ RGCs were significantly increased in the miR-21-5p agomir + AIH and S3I-201 + AIH groups. (**J**,**K**) Quantitative analysis of TUNEL^+^ apoptotic RGCs and RBPMS^+^ surviving RGCs, respectively (** *p* < 0.01, *** *p* < 0.001; one-way ANOVA, *n* = 3).

**Figure A2:**
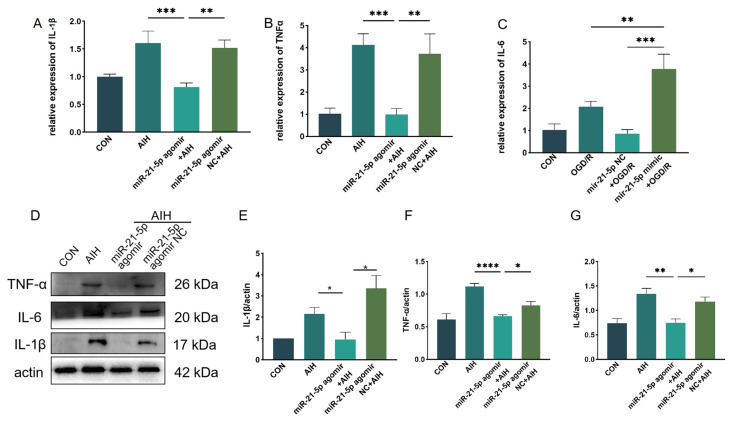
miR-21-5p attenuates STAT3-responsive inflammatory mediators in vivo after retinal I/R (AIH model). (**A**–**C**) qRT-PCR of IL-1β (**A**), TNF-α (**B**) and IL-6 (**C**) mRNA in rat retinas from CON, AIH, miR-21-5p agomir NC + AIH, and miR-21-5p agomir + AIH groups (3 d after AIH). Values were normalized to β-actin and calculated by the 2^^−ΔΔCt^ method. (**D**) Representative same-membrane immunoblots of IL-1β, TNF-α and IL-6 (sequentially reprobed after stripping) with ACTB as the loading control. (**E**–**G**) Densitometric quantification of protein levels for IL-1β (**E**), TNF-α (**F**) and IL-6 (**G**), normalized to ACTB. Data are presented as mean ± SD (mRNA: n = 3 independent biological samples per group; protein: n = 3 blots from independent animals). Statistics: one-way ANOVA with Bonferroni post hoc tests; * *p* < 0.05, ** *p* < 0.01, *** *p* < 0.001, **** *p* < 0.0001 vs. indicated com-parisons.

In vivo effects of miR-21-5p on retinal I/R injury. Rats received intravitreal injections of miR-21-5p agomir, scramble agomir (miR-21-5p agomir NC), or the STAT3 inhibitor S3I-201, followed by induction of the AIH model. After 3 days, eyeballs were enucleated, and retinas were dissected for analysis. WB analysis revealed that protein levels of STAT3 and p-STAT3 were significantly lower in the miR-21-5p agomir + AIH group compared with the AIH and NC + AIH groups (Figure 5A–C). Similar reductions in STAT3 and p-STAT3 expression were observed in the S3I-201 + AIH group. In addition, both mRNA and protein levels of STAT3 downstream inflammatory mediators, including IL-1β, TNF-α, and IL-6, were decreased in the miR-21-5p agomir + AIH group (Figure A2).

**Figure A3:**
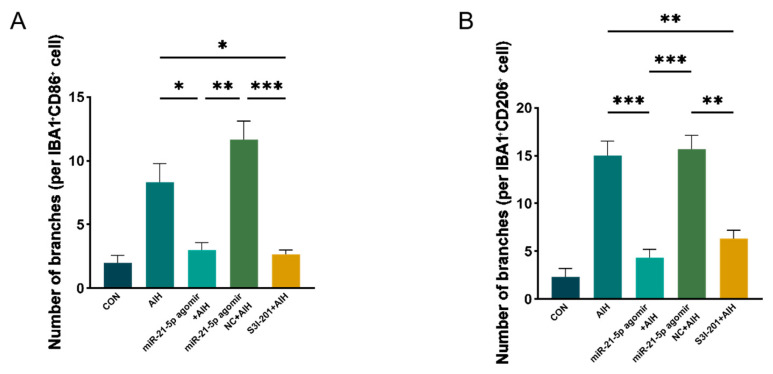
Branching of double-positive retinal microglia assessed by skeleton analysis. (**A**) IBA1^+^/CD86^+^ microglia (retina, in vivo). Double-positive cells were identified by IBA1/CD86 colocalization. Single-cell ROIs were binarized and skeletonized; the number of branches per cell was obtained with Analyze Skeleton (2D/3D) and averaged per animal. AIH increased the number of branches per cell compared with CON, and the agomir NC + AIH group showed a similar increase. In contrast, both miR-21-5p agomir + AIH and S3I-201 + AIH reduced branching toward CON levels. (**B**) IBA1^+^/CD206^+^ microglia analyzed with the same pipeline showed a concordant pattern. Bars represent mean ± SD (animals as the statistical unit, n = 3 per group). One-way ANOVA followed by Bonferroni post hoc test was used; * *p* < 0.05 ** *p* < 0.01, *** *p* < 0.001.

Immunofluorescence staining showed that the proportion of IBA1^+^CD86^+^ (M1-like) microglia was reduced and that of IBA1^+^CD206^+^ (M2-like) microglia was increased in both the miR-21-5p agomir + AIH and S3I-201 + AIH groups compared with the AIH and NC groups (Figure 5D–G). Using skeleton analysis on IBA1^+^ microglia, the AIH group displayed a marked increase in the number of branches per cell, whereas both the miR-21-5p agomir + AIH and S3I-201 + AIH groups significantly reduced the number of branches per cell toward CON levels (Figure A3). However, the soma area of microglia did not show a significant difference among groups. Cell survival assays demonstrated that the number of TUNEL^+^ RGCs was significantly reduced and the number of RBPMS^+^ RGCs was increased in the miR-21-5p agomir + AIH and S3I-201 + AIH groups relative to the AIH and NC groups (Figure 5H–K). Together, these in vivo experiments demonstrate that miR-21-5p overexpression attenuates retinal I/R injury by downregulating STAT3 signaling, inhibiting M1 polarization of microglia, and protecting RGCs from apoptosis.

## 4. Discussion

Our data suggest that miR-21-5p mitigates retinal I/R injury by downregulating STAT3, which in turn inhibits M1-like and favors M2-like microglial polarization, ultimately limiting loss of RGCs. Collectively, these results support a critical role of miR-21-5p in tuning microglial polarization and neuroinflammation, pointing to the miR-21-5p/STAT3 axis as a potential therapeutic target in ischemic retinal pathology.

Accumulating evidence indicates that miRNAs serve as key regulators of neuroinflammatory processes [44]. miR-21 is a highly conserved miRNA that is widely expressed in the brain and actively involved in CNS diseases [45,46,47]. miR-21 exerts neuroprotective effects in models of cerebral ischemia, subarachnoid hemorrhage (SAH), and traumatic brain injury (TBI), primarily by targeting p53, PTEN, or Rab11a and modulating the PI3K/AKT and Bcl-2/Bax pathways, thereby suppressing neuronal apoptosis and oxidative stress [19,48,49]. In retinal injury, a study found that increasing endogenous miR-21 levels can alleviate photoreceptor apoptosis and attenuate retinal structural and functional degeneration in an N-methyl-N-nitrosourea (MNU)-induced photoreceptor loss model [50]. Another study reported that miR-21-5p also exerts anti-inflammatory effects in retinal I/R injury by rebalancing NLRP3/6 inflammasome function and inhibiting microglial pyroptosis [4]. In this study, retinal miR-21-5p was downregulated in AIH rats, and miR-21-5p intervention mitigated AIH-induced retinal injury. Consistently, our data show that miR-21-5p overexpression enhances RGCs survival in I/R retinas and under OGD/R conditions. Together, our results indicate that increasing miR-21-5p may exert neuroprotective effects in retinal I/R pathology. However, chronic ocular hypertension (OHT)-induced glaucoma models do not show a clear upregulation of miR-21-5p [51,52]. The divergence may stem from differences in injury context: acute I/R entails robust inflammatory cascades and apoptotic responses, whereas chronic glaucoma is dominated by sustained mechanical loading and progressive neurodegeneration [53,54]. In light of our data, miR-21-5p may preferentially confer benefit in acute ischemic settings.

Microglial polarization critically shapes neuroinflammatory outcomes. The M1 phenotype produces pro-inflammatory cytokines (e.g., TNF-α, IL-1β) that aggravate neuronal damage, while the M2 phenotype secretes IL-4, IL-10, and neurotrophic factors, fostering tissue repair [6]. Microglial polarization plays a critical role in neuroinflammation, tissue repair, and neurodegenerative diseases, and its regulation involves multiple factors and pathways [55,56,57,58]. miRNAs critically shape microglial polarization. Notably, miR-9-5p targets and downregulates suppressor of cytokine signaling 2 (SOCS2), relieving its inhibitory constraint on JAK/STAT3 signaling, which in turn drives M1 microglial polarization [59]. By inhibiting the TLR4/NF-κB pathway and activating the PI3K/AKT pathway, miR-216a-5p promotes the transition of microglia from a pro-inflammatory M1 phenotype to an anti-inflammatory M2 phenotype [60]. Across in vivo and in vitro settings, upregulation of miR-21-5p attenuated M1 and favored M2 microglial polarization under I/R or OGD/R conditions, with concomitant reductions in pro-inflammatory mediators and increases in anti-inflammatory mediators, which collectively enhanced the survival of RGCs. In line with our findings, previous reports indicate that miR-21 attenuates inflammatory responses in retinal I/R through PDCD4 or TLR4-dependent mechanisms, and in brain ischemia fosters M2 polarization of microglia with concomitant neuroprotection by engaging the PI3K/AKT signaling axis [61,62,63]. Collectively, these data suggest that miR-21 may coordinately modulate microglial polarization through multiple targets, representing a promising target for regulating microglial polarization in retinal I/R injury and offering a new avenue for retinal protection.

miR-21-5p regulates several validated targets—BTG2, MELK, PDCD4, and STAT3—thereby influencing apoptosis and ferroptosis [64,65,66,67]. Notably, STAT3 is a key mediator of neuroinflammation, typically activated within the JAK/STAT axis to drive inflammatory gene expression [68]. Bioinformatic screening (TargetScan) coupled with dual-luciferase reporter assays, WB, and qRT-PCR demonstrated that STAT3 is a direct effector target of miR-21-5p. In the context of retinal I/R, enforced miR-21-5p expression reduced p-STAT3 and downregulated STAT3-responsive pro-inflammatory cytokines (TNF-α, IL-1β, IL-6), corroborating previously published findings [69,70]. STAT3 is a pleiotropic transcription factor that, upon JAK-mediated phosphorylation, translocates to the nucleus to regulate downstream gene expression, thereby participating in immune responses, cell growth, and apoptosis [71,72]. Our data indicate that pharmacologic inhibition of STAT3 dampens M1-like and favors M2-like microglial polarization, with concomitant decreases in pro-inflammatory and increases in anti-inflammatory mediators. Consistently, in vivo STAT3 inhibition under I/R conditions was associated with improved RGC survival. We further provide evidence that miR-21-5p directly targets STAT3, leading to lowered STAT3 phosphorylation and repression of STAT3-responsive inflammatory mediators, thereby mitigating retinal I/R damage. Consistent with our findings, previous studies have shown that reducing STAT3 activity in in vitro and in vivo models markedly attenuates microglia-mediated neuroinflammation and suppresses the expression of pro-inflammatory cytokines such as IL-1β and TNF-α [73]. Complementing these disease-context data, recent chemical-biology advances have yielded azetidine-based covalent STAT3 inhibitors that selectively and irreversibly engage STAT3 and demonstrate in vivo efficacy [33]. Structure–activity work further identifies the azetidine ring and salicylic-acid/bioisosteric motifs as key determinants of STAT3 engagement [34]. Meanwhile, in a neonatal mouse hypoxia–ischemia (HI) model, Xin et al. reported that elevating miR-21a-5p (the murine ortholog of miR-21-5p) downregulated p-STAT3 and biased microglia toward an M2-like phenotype, thereby conferring neuroprotection [74]. Taken together, lines of evidence from miRNA-based modulation and pharmacologic suppression of the signaling cascade support STAT3 as a pivotal node that orchestrates microglial polarization and neuroinflammation.

Our investigation centers on miR-21-5p in microglia, and we did not comprehensively assess its effects in astrocytes or neurons. Because miR-21-5p may exhibit cell-type-specific targeting, future studies combining single-cell transcriptomic/proteomic approaches with protein–protein interaction mapping are warranted to resolve its intercellular specificity and STAT3-related activation mechanisms.

Taken together, our data indicate that miR-21-5p, via STAT3 downregulation, suppresses M1-like while favoring M2-like microglial polarization, thereby dampening inflammatory responses and preserving RGCs in retinal I/R injury. This work strengthens the mechanistic framework for miRNA-driven regulation in ischemic retinal pathology and highlights STAT3 as a promising, potentially translatable therapeutic target.

## 5. Conclusions

In conclusion, this research indicates that miR-21-5p mitigates retinal I/R injury by downregulating STAT3, which suppresses M1-like and favors M2-like microglial polarization, thereby dampening inflammatory responses and limiting loss of RGCs.

## Data Availability

The data supporting the findings of this study are available from the corresponding author upon reasonable request.

## Abbreviations

The following abbreviations are used in this manuscript:

I/R	Ischemia-reperfusion
RGCs	Retinal ganglion cells
AIH	Acute intraocular hypertension
OGD/R	Oxygen and glucose deprivation/reperfusion
MCM	Microglial-conditioned media
RM	Rat Microglia
STAT3	Signal Transducer and Activator of Transcription 3
Arg-1	Arginase-1
IL-1β	Interleukin-1 beta
LPS	Lipopolysaccharide
TLR	Toll-like receptor
IFN-γ	Interferon-gamma
ROS	Reactive Oxygen Species
ECM	Extracellular Matrix
TNF-α	Tumor Necrosis Factor-alpha
3′-UTR	3′ Untranslated Region
TM	Trabecular Meshwork
